# Epidemiology and prognostic factors of nosocomial candidemia in Northeast Brazil: A six-year retrospective study

**DOI:** 10.1371/journal.pone.0221033

**Published:** 2019-08-22

**Authors:** Mariana Araújo Paulo de Medeiros, Ana Patrícia Vieira de Melo, Aurélio de Oliveira Bento, Luanda Bárbara Ferreira Canário de Souza, Francisco de Assis Bezerra Neto, Jarmilla Bow-Ltaif Garcia, Diana Luzia Zuza-Alves, Elaine Cristina Francisco, Analy Salles de Azevedo Melo, Guilherme Maranhão Chaves

**Affiliations:** 1 Laboratory of Medical and Molecular Mycology, Department of Clinical and Toxicological Analysis, Federal University of Rio Grande do Norte, Natal city, Rio Grande do Norte State, Brazil; 2 Special Mycology Laboratory, Department of Medicine, Federal University of Sao Paulo, São Paulo City, São Paulo State, Brazil; Vita Salute University of Milan, ITALY

## Abstract

Candidemia has been considered a persistent public health problem with great impact on hospital costs and high mortality. We aimed to evaluate the epidemiology and prognostic factors of candidemia in a tertiary hospital in Northeast Brazil from January 2011 to December 2016. Demographic and clinical data of patients were retrospectively obtained from medical records and antifungal susceptibility profiling was performed using the broth microdilution method. A total of 68 episodes of candidemia were evaluated. We found an average incidence of 2.23 episodes /1000 admissions and a 30-day mortality rate of 55.9%. The most prevalent species were *Candida albicans* (35.3%), *Candida tropicalis* (27.4%), *Candida parapsilosis* (21.6%) and *Candida glabrata* (11.8%). Higher mortality rates were observed in cases of candidemia due to *C*. *albicans* (61.1%) and *C*. *glabrata* (100%), especially when compared to *C*. *parapsilosis* (27.3%). Univariate analysis revealed some variables which significantly increased the probability of death: older age (*P* = 0.022; odds ratio [OR] = 1.041), severe sepsis (*P* < 0.001; OR = 8.571), septic shock (*P* = 0.035; OR = 3.792), hypotension (*P* = 0.003; OR = 9.120), neutrophilia (*P* = 0.046; OR = 3.080), thrombocytopenia (*P* = 0.002; OR = 6.800), mechanical ventilation (*P* = 0.009; OR = 8.167) and greater number of surgeries (*P* = 0.037; OR = 1.920). Multivariate analysis showed that older age (*P* = 0.040; OR = 1.055), severe sepsis (*P* = 0.009; OR = 9.872) and hypotension (*P* = 0.031; OR = 21.042) were independently associated with worse prognosis. There was no resistance to amphotericin B, micafungin or itraconazole and a low rate of resistance to fluconazole (5.1%). However, 20.5% of the *Candida* isolates were susceptible dose-dependent (SDD) to fluconazole and 7.7% to itraconazole. In conclusion, our results could assist in the adoption of strategies to stratify patients at higher risk for developing candidemia and worse prognosis, in addition to improve antifungal management.

## Introduction

Candidemia, or the bloodstream infection (BSI) caused by *Candida* species, is a subset of invasive candidiasis (IC) with increased incidence over the last few decades, considered a persistent public health problem with great impact on health care-associated costs and high crude (35% to 75%) and attributable mortality, despite advances achieved in diagnosis and treatment [1–6].

*Candida* species are generally referred as the fourth leading cause of nosocomial BSI in the United States (US), accounting for 8 to 10% of all hospital-acquired BSIs [1–3]. Recently, a study encompassing several US states reported *Candida* spp. as the most prevalent pathogens obtained from nosocomial BSIs, even overcoming some common bacterial species [7].

At least 15 different *Candida* spp. have been reported to cause human invasive infections. Nevertheless, more than 90% of them are caused by five main species, as follows: *Candida albicans*, *Candida glabrata*, *Candida parapsilosis*, *Candida tropicalis* and *Candida krusei* [2, 3, 8, 9]. The distribution of *Candida* spp. causing candidemia presents temporal and geographic variation, alongside the considerable influence of patient characteristics, antifungal stewardship and clinical practices [2, 3, 8]. Although *C*. *albicans* remains the most frequently isolated species from *Candida* BSI episodes, its incidence has recently decreased [2, 3, 8, 10].

Most candidemia predisposing factors are very common among critically ill patients in the ICU. This fact, together with the delay and lack of sensitivity of diagnostic tools, impair the prompt recognition and treatment of this infection [2, 6]. Moreover, antifungal susceptibility profiling may vary according to each *Candida* species and even within strains of the same species, whilst the development of microbial resistance may occur to any class of antifungal agents, making the management of candidemia even more difficult [2, 11].

Since the indiscriminate use of antifungals can generate great economic and ecological impact, antifungal prophylaxis and empirical treatment should be considered only in high-risk patients selected through strategies such as the colonization index, Candida score, and predictive rules based on combinations of risk factors [2, 5, 6, 12, 13, 14].

Considering the scarcity of *Candida* BSIs studies conducted in Northeast Brazil (Brazil’s lowest income region) and the relevance of the knowledge of local peculiarities to assist the optimization of strategies for prevention and treatment of infections, we aimed to evaluate the epidemiology of candidemia and risk factors associated with mortality in a tertiary hospital in this Brazilian region over 6 years.

## Materials and methods

### Study design

This is a retrospective, single-center, observational cohort study conducted at Onofre Lopes Hospital (Natal city, Brazil), a tertiary University Hospital with 248 beds. All patients who developed candidemia during a 6-year period (from January 2011 to December 2016) were included in the study. Candidemia or *Candida* BSI was defined as at least one positive blood culture for *Candida* spp. in patients hospitalized for more than 48 h. Only the first episode of candidemia was recorded for each patient. Therefore, *Candida* BSI episodes which occurred before 48 hours of hospitalization or represented relapses were excluded. Demographic and clinical data were collected from medical records within the preceding 30 days from the onset of *Candida* BSI (defined as the day of first *Candida* spp. positive blood culture) up to a 30-day follow-up period, except for data on surgery (collected up to 3 months before the onset of candidemia). Clinical data included vital signs, blood count, other infections/positive cultures, underlying conditions, predisposing factors for candidemia, previous exposure to antifungals, clinical management and outcome (survival or death). Vital signs were classified according to the parameters established in the literature [15, 16] together with the medical interpretation in the patients' records. Classification of blood cell counts were based on the reference ranges defined locally by the hospital laboratory. Sepsis, severe sepsis and septic shock were defined according to Angus and van der Poll [17]. Crude mortality rate was calculated at 7 and 30 days from candidemia onset. The following antifungal dosages were considered adequate: fluconazole (FLU) 400 mg/day, amphotericin B deoxycholate (AMB) 0.5–1.0 mg/kg/day, amphotericin B lipid complex (ABLC) 3.0–5.0 mg/kg/day, caspofungin (CPF) 50 mg/day, micafungin (MCF) 100 mg/day, anidulafungin (ADF) 100 mg/day [18].

### Ethics

This study was approved by the Local Research Ethics Committee (“Comitê de Ética em Pesquisa da Liga Norte Riograndense Contra o Câncer”) under the protocol number 042/042/2012. Written patient consent was not required because of the observational nature of the study.

### Laboratory procedures

Blood samples were processed using the Bact/Alert system (BioMérieux, France). All positive cultures were inoculated onto the surface of Sheep Blood Agar and incubated at 30°C for 48–96 h. Yeast growth was confirmed by Gram staining and the initial identification was performed at the referred hospital with the Vitek 2 Compact YST system (BioMérieux, France), according with manufacturer’s instructions. The strains were sent to the Laboratory of Medical and Molecular Mycology, Department of Clinical and Toxicological Analysis, Federal University of Rio Grande do Norte and a confirmation in identification was performed according to classical methods [19]. Of note, accurate identification was also performed using MALDI-TOF [20] when necessary. Unfortunately, some strains were not identified at the species level due to limitations of the initial screening performed at the hospital microbiology laboratory and lack of viability/or availability of some strains for further analysis. Antifungal susceptibility to amphotericin B (Sigma Chemical Corporation, St. Louis, MO, USA), fluconazole (Pfizer Incorporated, New York, NY, USA), itraconazole (Pfizer Incorporated, New York, NY, USA) and micafungin (Merck, Rahway, NJ, USA) was performed using the broth microdilution method according to the Clinical and Laboratory Standards Institute (CLSI) document M27-A3 [21]. The reference strains *C*. *parapsilosis* ATCC 22019 and *C*. *krusei* ATCC 6258 were used as quality controls. Minimum inhibitory concentration (MIC) values were interpreted according to the current clinical breakpoints suggested by CLSI for the most common species of *Candida* [22, 23].

### Statistical analysis

Continuous variables were expressed as mean ± standard deviation (SD) and compared using Student *t* test or Mann-Whitney test. Categorical variables were expressed as frequencies and percentages and compared using Chi- square (*X*^2^) test or Fisher’s exact test, as appropriate. Logistic regression analysis was performed with variables that presented *P* ≤ 0.1 in the comparisons of groups to identify possible risk factors associated with mortality at 30 days after candidemia. Variables of clinical relevance and with sample size ≥60 found to be significant in the univariate analysis were included in a multivariate logistic model. All tests were 2-tailed, and a *P*-value <0.05 was determined to represent statistical significance. Statistical analyses were performed using the Statistical Package for the Social Sciences (SPSS) software, version 20 (IBM SPSS, Chicago, IL, USA).

## Results

**Fig 1:**
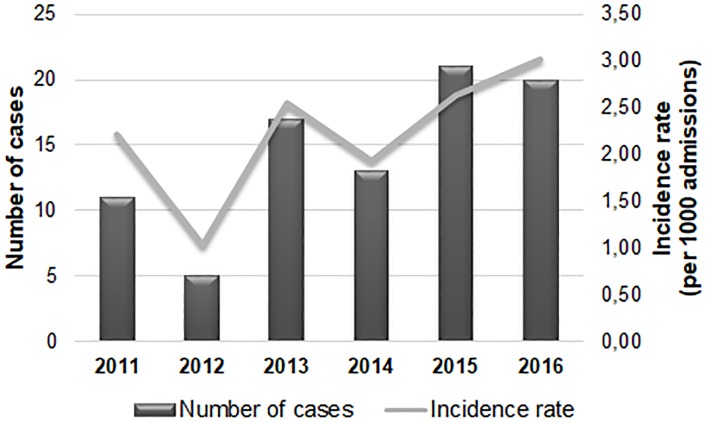
Number of cases and incidence rate of candidemia observed during a 6-year period in a tertiary hospital in Northeast Brazil.

A total of 87 patients out of 37,768 admitted to the study hospital between 2011 and 2016 had at least one episode of candidemia. However, 19 individuals were excluded (16 patients who had candidemia before 48 h of hospitalization and 3 patients with no medical records). The mean incidence of candidemia cases was 2.23/1000 admissions, ranging from 1.03 to 3.02 throughout each different year of study, with a trend to increase from 2014–2016 (Fig 1).

**Fig 2:**
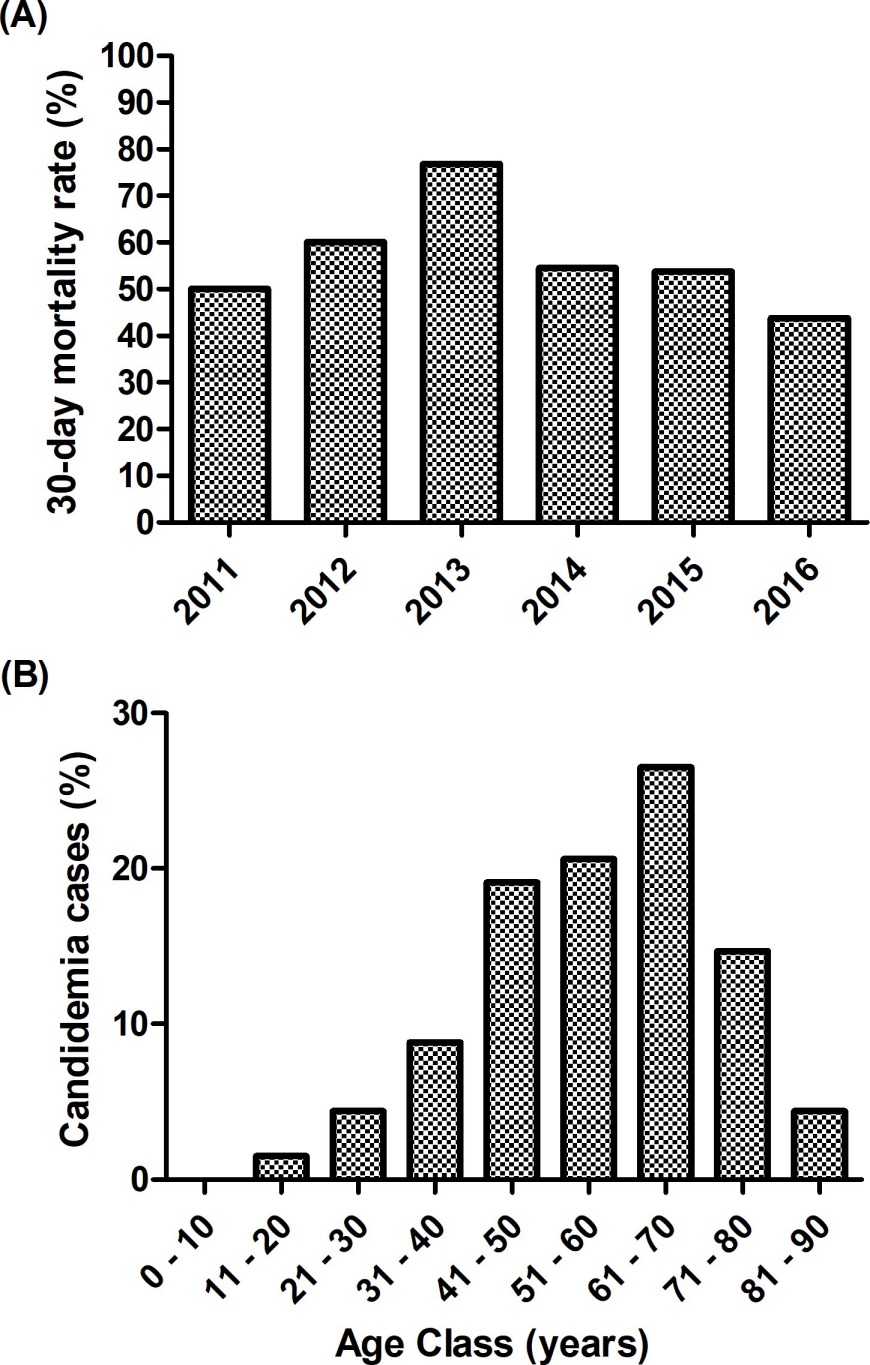
**30-day mortality rate during a 6-year period** (A) and age class distribution (B) of patients with candidemia in a tertiary hospital in Northeast Brazil.

The 7-day and 30-day mortality rates were 33.8% (23/68) and 55.9% (38/68), respectively. The 30-day mortality rate was much higher in the ICU (70.8%, 17/24) compared to other sectors of the hospital (20/40; 50%). Over the 6 years of the study, the 30-day mortality rate ranged from 43.8% (7/16) in 2016 to 76.9% (10/13) in 2013, with a trend to increase between 2011 and 2013; and decrease between 2013 and 2016 (Fig 2A).

Positive cultures for bacteria were obtained from 73.5% (50/68) of the patients, including blood cultures (32/68; 47.1%). Mixed bacterial and yeast bloodstream infection occurred on the day of candidemia onset in 8 cases (8/68; 11.8%). Other yeast-positive cultures were obtained from 47.1% (32/68) of the patients, comprising sterile (24/68; 35.3%) and non-sterile body sites (16/68; 23.5%).

At the onset of candidemia, 37.5% (24/64) of the patients were in the ICU, 23.4% (15/64) in internal medicine wards, 18.8% (12/64) in surgical wards, 7.8% (5/64) in cardiovascular wards (Table 1), 7.8% (5/64) in isolation wards, 3.1% (2/64) in transplantation wards and 1.6% (1/64) in oncohematology wards.

**Table 1:** Demographic characteristics and underlying conditions of patients with candidemia, including comparison between subgroups according to the outcome.

Characteristics of patients, N (%)	All patients (N = 68)	30-day outcome		*P*-value*
		Survival (N = 30)	Death (N = 38)	
**Gender (male)**	27 (39.7)	10 (33.3)	17 (44.7)	0.340
**Age (years; mean ± SD)**	56.0 ± 15.5	51.0 ± 17.0	60 ± 13.2	**0.017**
**Underlying Conditions**				
**Cancer**	28 (42.4)	10 (33.3)	18 (50.0)	0.173
**Cardiovascular Disease**	49 (72.1)	21 (70.0)	28 (73.7)	0.737
**Gastrointestinal Disease**	25 (36.8)	9 (30.0)	16 (42.1)	0.304
**Renal Failure**	35 (51.5)	12 (40.0)	23 (60.5)	0.093
**Chronic Renal Failure**	15 (23.1)	7 (58.3)	8 (40.0)	0.964
**Acute Renal Failure**	17 (26.2)	5 (41.7)	12 (60.0)	0.107
**Lung Disease**	5 (7.4)	1 (3.3)	4 (10.5)	0.374
**Diabetes Mellitus**	22 (59.5)	11 (55.0)	11 (64.7)	0.549
**Obesity**	4 (5.9)	1 (3.3)	3 (7.9)	0.624

Some information was missing in patients' records; therefore the valid N varies according to the variable.

* Student *t* Test/Mann-Whitney Test (continuous data) or Chi-Square Test/Fisher Exact Test (categorical data).

*Candida albicans* was the most prevalent species obtained from blood cultures, accounting for 35.3% (18/51) of candidemia episodes, followed by *Candida tropicalis* (14/51; 27.4%), *Candida parapsilosis* (11/51; 21.6%), *Candida glabrata* (6/51; 11.8%) and other less common species (2/51; 3.9%), including one episode caused by *Candida lusitaniae* (2%) and another by *Kodamaea ohmeri* (2%).

Tables 1 to 4 present the main demographic and clinical characteristics of all the patients included in the present study, classified according to the outcome of candidemia after 30 days (survival or death).

**Table 2:** Clinical condition of patients on candidemia onset, including comparison between subgroups according to the outcome.

Characteristics of patients, N (%)		All patients (N = 68)	30-day outcome		*P*-value*
			Survival (N = 30)	Death (N = 38)	
**Sepsis**		53 (77.9)	20 (66.7)	33 (86.8)	**0.046**
**Severe Sepsis**		29 (42.6)	5 (16.7)	24 (63.2)	**<0.001**
**Septic Shock**		18 (26.5)	4 (13.3)	14 (36.8)	**0.029**
**Vital Signs**					
**Fever**		29 (44.6)	16 (53.3)	13 (37.1)	0.191
**Heart Rate**	**Bradycardia**	2 (3.3)	1 (3.8)	1 (2.9)	0.936
	**Normocardia**	20 (32.8)	9 (34.6)	11 (31.4)	
	**Tachycardia**	39 (63.9)	16 (61.5)	23 (65.7)	
**Respiratory Frequency**	**Bradypnea**	0	0	0	0.609
	**Eupnea**	21 (32.3)	10 (35.7)	11 (29.7)	
	**Tachypnea**	44 (67.7)	18 (64.3)	26 (70.3)	
**Blood Pressure**	**Hypotension**	24 (40.0)	5 (19.2)	19 (55.9)	**0.006**
	**Normotension**	19 (31.7)	9 (34.6)	10 (29.4)	
	**Hypertension**	17 (28.3)	12 (46.2)	5 (14.7)	
**Blood Count**					
**Blood Leucocyte Count**	**Leukopenia**	5 (8.5)	1 (4.2)	4 (11.4)	0.224
	**Normal**	20 (33.9)	11 (45.8)	9 (25.7)	
	**Leukocytosis**	34 (57.6)	12 (50.0)	22 (62.9)	
**Blood Neutrophil Count**	**Neutropenia**	3 (5.1)	0	3 (8.6)	**0.042**
	**Normal**	24 (40.7)	14 (58.3)	10 (28.6)	
	**Neutrophilia**	32 (54.2)	10 (41.7)	22 (62.9)	
**Blood Lymphocyte Count**	**Lymphopenia**	18 (30.5)	7 (29.2)	11 (31.4)	0.175
	**Normal**	33 (55.9)	16 (66.7)	17 (48.6)	
	**Lymphocytosis**	8 (13.6)	1 (4.2)	7 (20.0)	
**Anemia**		55 (91.7)	23 (92.0)	32 (91.4)	0.937
**Blood Platelet Count**	**Thrombocytopenia**	27 (46.6)	5 (21.7)	22 (62.9)	
	**Normal**	28 (48.3)	17 (73.9)	11 (31.4)	**0.006**
	**Thrombocytosis**	3 (5.2)	1 (4.3)	2 (5.7)	

Candidemia onset was defined as the day of first positive blood culture for *Candida* species. Some information was missing in patients' records; therefore the valid N varies according to the variable.

* Student *t* Test/Mann-Whitney Test (continuous data) or Chi-Square Test/Fisher Exact Test (categorical data).

**Table 3:** Predisposing factors for *Candida* bloodstream infection and other characteristics of patients with candidemia, including comparison between subgroups according to the outcome.

Characteristics of patients, N (%)	All patients (N = 68)	30-day outcome		*P*-value*
		Survival (N = 30)	Death (N = 38)	
**Medical Devices**				
**Central Venous Catheter (CVC)**	54 (79.4)	26 (86.7)	28 (73.7)	0.189
**CVC removal within 48 hours**	12 (23.1)	8 (34.8)	4 (13.8)	0.074
**Total Parenteral Nutrition**	23 (33.8)	11 (36.7)	12 (31.6)	0.660
**Mechanical Ventilation (MV)**	22 (32.4)	9 (30.0)	13 (34.2)	0.712
**MV on candidemia onset**	16 (23.5)	2 (6.7)	14 (36.8)	**0.004**
**Other Features**				
**Previous Bacteremia**	19 (27.9)	9 (30.0)	10 (26.3)	0.737
**Previous use of antibacterial agents**	66 (97.1)	29 (96.7)	37 (97.4)	0.865
**Nº of antibacterial agents used previously (mean ± SD)**	3.4 ± 1.6	3.0 ± 1.2	3.7 ± 1.8	0.057
**Post use of antibacterial agents**	62 (92.5)	25 (86.2)	37 (97.4)	0.158
**Corticosteroid Therapy**	38 (55.9)	17 (56.7)	21 (55.3)	0.908
**Other immunosuppressants**	4 (5.9)	3 (10.0)	1 (2.6)	0.314
**Chemotherapy**	5 (7.4)	2 (6.7)	3 (7.9)	0.847
**Hemodialysis**	19 (27.9)	10 (33.3)	9 (23.7)	0.379
**Surgery**	38 (55.9)	20 (66.7)	18 (47.4)	0.112
**Number of surgeries (mean ± SD)**	2.1 ± 1.4	1.7 ± 0.7	2.7 ± 1.7	0.105
**Abdominal Surgery**	31 (45.6)	16 (53.3)	15 (39.5)	0.255
**Kidney Transplantation**	3 (4.4)	3 (10.0)	0	0.081

Characteristics of patients with unspecified temporal relation or named as “previous” were collected from medical records only within the preceding 30 days from the candidemia onset (defined as the day of first positive blood culture for *Candida* species), except for data on surgery (collected up to 3 months before candidemia onset). Characteristics of patients named as “post” were collected from medical records up to a 30-day follow-up period from the candidemia onset. Some information was missing in patients' records; therefore the valid N varies according to the variable.

* Student *t* Test/Mann-Whitney Test (continuous data) or Chi-Square Test/Fisher Exact Test (categorical data).

**Table 4:** Antifungal stewardship in patients with candidemia, including comparison between subgroups according to the outcome.

Characteristics of patients, N (%)	All patients (N = 68)	30-day outcome		*P*-value*
		Survival (N = 30)	Death (N = 38)	
**Previous exposure to antifungals**	11 (16.2)	4 (13.3)	7 (18.4)	0.572
**Antifungal Treatment**	41 (61.2)	20 (69.0)	21 (55.3)	0.254
Timing of antifungal administration^**^ **(days; mean ± SD)**	5.0 ± 6.0	5.8 ± 7.3	4.2 ± 4.3	0.423
**Adequate antifungal dosage**	28 (68.3)	13 (65.0)	15 (71.4)	0.658

Previous exposure to antifungals was collected from medical records within the preceding 30 days from the candidemia onset (defined as the day of first positive blood culture for *Candida* species). Some information was missing in patients' records; therefore the valid N varies according to the variable.

* Student *t* Test/Mann-Whitney Test (continuous data) or Chi-Square Test/Fisher Exact Test (categorical data).

^**^Timing of antifungal administration: Interval between candidemia onset and initiation of antifungal treatment.

The main predisposing factors found were the previous use of antibacterial agents (66/68; 97.1%), the presence of CVC (54/68; 79.4%), corticosteroid therapy (38/68; 55.9%) and surgery (38/68; 55.9%; Table 3).

Of the 68 patients included in the study, 19 came from another hospital at admission (27.9%), 41 were female (60.3%) with a mean age of 56.0 ± 15.5 years (Table 1), mean hospital length of stay (LOS) of 63.9 ± 50.5 days and mean time between admission and development of candidemia of 35.6 ± 32.2 days.

The predominant age class ranged from 61 to 70 years (18/68; 26.5%; Fig 2B). It is worth mentioning that 45.6% (31/68) of the patients were elderly (aged between 61 and 90 years); while only one 12-year-old child was enrolled in the study since our hospital did not have a pediatric ward (Fig 2B).

The most prevalent underlying conditions were cardiovascular disease (49/68; 72.1%), diabetes mellitus (22/37; 59.5%) and renal failure (35/68; 51.5%; Table 1). Other important comorbidities were cancer (28/66; 42.4%), including three cases of hematological malignancy (3/28; 10.7%); and gastrointestinal disease (25/68; 36.8%; Table 1).

Most of the patients presented sepsis (53/68; 77.9%) and, to a lesser extent, severe sepsis (29/68; 42.6%) and/or septic shock (18/68; 26.5%) at the time of candidemia (Table 2). Among the 53 patients who developed sepsis, 19 of them had only sepsis (19/68; 27.9%), 16 developed severe sepsis (16/68; 23.5%), five developed septic shock (5/68; 7.4%), while 13 patients had both severe sepsis and septic shock (13/68; 19.1%).

The use of antifungal agents before the onset of candidemia was observed in 16.2% (11/68) of the patients (Table 4), of which 63.6% (7/11) used fluconazole and 63.6% (7/11) used antifungal drugs for very short periods (1 to 5 days). The previous exposure to antifungals did not influence the isolation of NCAC species (*P* = 0.451).

Antifungal treatment was instituted in 61.2% of the patients (41/67; Table 4) and the most commonly used antifungal agent was fluconazole (38/41; 92.7%), alone (27/41; 65.9%) or in combination with other antifungal agents (11/41; 26.8%), mainly with amphotericin B deoxycholate (6/41; 14.6%). Echinocandins were used only in four patients among the group receiving treatment (9.8%).

Antifungal treatment did not influence the outcome of candidemia (*P* = 0.254; Table 4), and it is worth mentioning that 34.6% (9/26) of patients who were not treated survived.

**Fig 3:**
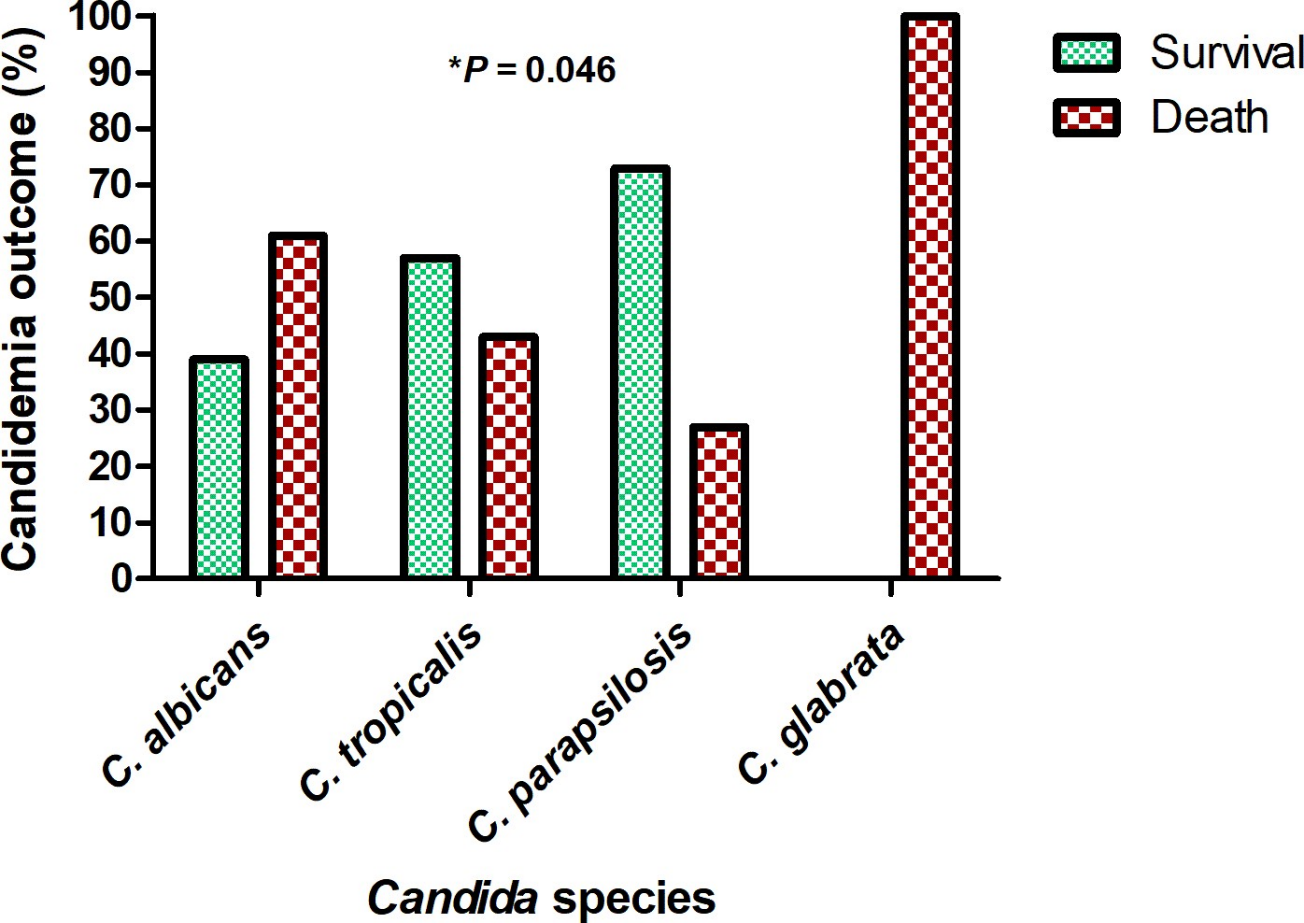
Outcome of candidemia according to the *Candida* species in a tertiary hospital in Northeast Brazil. * Chi- square (*X*^2^) test.

Compared to patients who survived, patients who died within 30 days after candidemia were older (*P* = 0.017), had a higher frequency of sepsis (*P* = 0.046), severe sepsis (*P* <0.001), septic shock (*P* = 0.029), hypotension (*P* = 0.006), neutrophilia (*P* = 0.042), thrombocytopenia (*P* = 0.006), the use of MV on candidemia onset (*P* = 0.004; Tables 1–3) and *C*. *albicans* and *C*. *glabrata* in blood cultures (*P* = 0.046; Fig 3).

The relationship between these variables and the outcome was confirmed in the univariate logistic regression, except for sepsis and *Candida* species isolated (Table 5). Some characteristics of the patients significantly increased the probability of death: older patients (*P* = 0.022; OR = 1.041), severe sepsis (*P* < 0.001; OR = 8.571), septic shock (*P* = 0.035; OR = 3.792), hypotension *vs*. hypertension (*P* = 0.003; OR = 9.120), neutrophilia (*P* = 0.046; OR = 3.080), thrombocytopenia (*P* = 0.002; OR = 6.800), MV on candidemia onset (*P* = 0.009; OR = 8.167) and greater number of surgeries (*P* = 0.037; OR = 1.920; Table 5).

**Table 5:** Univariate logistic regression analysis of risk factors for 30-day mortality.

Characteristics of patients	Univariate analysis		
	*P*-value	Odds ratio	95% CI
**Age (years; mean ± SD)**	**0.022**	1.041	1.006–1.078
**Sepsis**	0.053	3.300	0.985–11.052
**Severe Sepsis**	**<0.001**	8.571	2.675–27.470
**Septic Shock**	**0.035**	3.792	1.095–13.129
**Blood Pressure** **(Hypotension *vs*. Hypertension)**	**0.003**	9.120	2.172–38.296
**Blood Neutrophil Count** **(Neutrophilia *vs*. Normal count)**	**0.046**	3.080	1.022–9.284
**Blood Platelet Count** **(Thrombocytopenia *vs*. Normal count)**	**0.002**	6.800	1.983–23.314
***C*. *tropicalis* vs. *C*. *albicans***	0.308	0.477	0.115–1.976
***C*. *parapsilosis* vs. *C*. *albicans***	0.085	0.239	0.047–1.219
***C*. *glabrata* vs. *C*. *albicans***	0.999	-	-
**CVC removal within 48 hours**	0.083	0.300	0.077–1.169
**MV on candidemia onset**	**0.009**	8.167	1.684–39.598
**Nº of antibacterial agents used previously (mean ± SD)**	0.064	1.393	0.981–1.978
**Surgery**	0.114	0.450	0.167–1.212
**Number of surgeries (mean ± SD)**	**0.037**	1.920	1.041–3.544
**Kidney Transplantation**	0.999	-	-
**Renal Failure**	0.095	2.300	0.865–6.117
**Acute Renal Failure**	0.113	2.609	0.796–8.550

CI: confidence interval; CVC: central venous catheter; MV: mechanical ventilation.

Multivariate analysis included age, severe sepsis, septic shock, use of MV and blood pressure on candidemia onset (Table 6). Age (*P* = 0.040; OR = 1.055), severe sepsis (*P* = 0.009; OR = 9.872) and hypotension *vs*. hypertension (*P* = 0.031; OR = 21.042) were independently associated with higher probability of death (Table 6). It is worth mentioning that the probability of death increased about 10-fold in patients who had severe sepsis and 21-fold in patients with hypotension compared to those who had hypertension at the onset of candidemia.

**Table 6:** Multivariate logistic regression analysis of risk factors for 30-day mortality.

Characteristics of patients	Multivariate analysis^*^		
	*P*-value	Odds ratio	95% CI
**Age (mean ± SD)**	**0.040**	1.055	1.003–1.110
**Severe Sepsis**	**0.009**	9.872	1.776–54.880
**Septic Shock**	0.558	0.451	0.032–6.462
**Blood Pressure** **(Hypotension *vs*. Hypertension)**	**0.031**	21.042	1.318–336.004
**MV on candidemia onset**	0.353	2.613	0.344–19.869

^*^ *X*^2^(7) = 30.466; *P* < 0.001; R^2^ Nagelkerke = 0.540; Hosmer-Lemeshow Test *P* = 0.263; Specificity = 76.9%; Sensitivity = 84.8%; Accuracy = 81.4%. CI: confidence interval; MV: mechanical ventilation.

Table 7 shows the results of the *in vitro* activity of 4 systemically active antifungal agents against BSI isolates of *Candida* spp. All isolates tested were susceptible to amphotericin B and micafungin, while a few of them were resistant (2/39; 5.1%) and susceptible dose-dependent (SDD; 8/39; 20.5%) to fluconazole and SDD to itraconazole (3/39; 7.7%). There were two strains (an isolate of *C*. *albicans* and another of *C*. *tropicalis*) SDD to both fluconazole and itraconazole (2/39; 5.1%).

**Table 7:** Antifungal susceptibility test results for Candida spp. isolates.

Species / Antifungal agent	MIC (μg/ml)			Resistance N (%)	S-DD N (%)
	Range	MIC_50_	MIC_90_		
**All isolates tested (N = 39)**					
**Amphotericin B**	0.06–1.0	0.25	1.0	0	-
**Fluconazole**	0.125–64.0	1.0	2.0	2 (5.1)	8 (20.5)
**Itraconazole**	<0.03–0.25	0.03	0.06	0	3 (7.7)
**Micafungin**	<0.015–1.0	<0.015	0.03	0	-
***Candida albicans* (N = 13 tested)**					
**Amphotericin B**	0.125–1.0	0.25	0.5	0	-
**Fluconazole**	0.125–4.0	0.5	4.0	0	2 (15.4)
**Itraconazole**	0.03–0.25	0.06	0.125	0	2 (15.4)
**Micafungin**	<0.015–0.06	<0.015	0.03	0	-
***Candida tropicalis* (N = 12 tested)**					
**Amphotericin B**	0.06–1.0	0.25	1.0	0	-
**Fluconazole**	0.5–4.0	0.5	4.0	0	2 (16.7)
**Itraconazole**	0.03–0.125	0.03	0.06	0	1 (8.3)
**Micafungin**	<0.015–0.06	<0.015	0.03	0	-
***Candida parapsilosis* (N = 9 tested)**					
**Amphotericin B**	0.125–1.0	0.5	-	0	-
**Fluconazole**	0.125–16.0	0.5	-	1 (11.1)	0
**Itraconazole**	<0.03–0.03	<0.03	-	0	0
**Micafungin**	<0.015–1.0	0.03	-	0	-
***Candida glabrata* (N = 5 tested)**					
**Amphotericin B**	0.06–1.0	0.25	-	0	-
**Fluconazole**	0.5–64.0	1.0	-	1 (20)	4 (80)
**Itraconazole**	0.03–0.06	0.03	-	0	0
**Micafungin**	<0.015–0.06	<0.015	-	0	-

MIC: Minimum Inhibitory Concentration. MIC_50_ and MIC_90_: MIC required to inhibit 50% and 90% of the isolates, respectively. S-DD: Susceptible-Dose Dependent. Resistance breakpoints: fluconazole: MIC of ≥8 μg/ml; ≥64 μg/ml for *C*. *glabrata*; itraconazole: ≥1 μg/ml; amphotericin B: ≥2 μg/ml; micafungin: ≥1 μg/ml; ≥8 μg/ml for *C*. *parapsilosis*; ≥0.25 μg/ml for *C*. *glabrata*. S-DD breakpoints: fluconazole: MIC of 4 μg/ml; ≤32 μg/ml for *C*. *glabrata*; itraconazole: 0.25–0.5 μg/ml.

## Discussion

The overall incidence rate of candidemia observed in our study (2.23 episodes per 1000 admissions) was close to the findings of Brazilian multicenter studies (2.42 to 2.49/1000 admissions) [24, 25] and also those reported in the US (1.9 to 2.4/1000 admissions) [2], but higher than the rates reported in a multicenter study in Latin America (1.18/1000 admissions) [26], in several European countries (0.23 to 1.5/1000 admissions) [27–34] and in a recent study conducted in Japan (0.056/1000 admissions) [35].

Compared to other studies around the world [32, 35, 36, 37], our patient’s mortality rate (55.9%) is higher, corroborating other Brazilian studies, ranging from 54 to 72.2% [24, 38, 39].

The distribution of *Candida* species observed in our study is consistent with other studies conducted in Brazil and Latin America, showing a relatively lower prevalence of *C*. *albicans* (although it is still the most prevalent species) and a higher prevalence of *C*. *parapsilosis* and *C*. *tropicalis* among the NCAC species (alternating between second and third places), and *C*. *glabrata* as the fourth most prevalent species [24, 26, 38, 39]; whereas in the US and several other European countries *C*. *glabrata* appears generally as the second most prevalent species [8].

*C*. *albicans* and *C*. *glabrata* were the species most associated with mortality, especially when compared to *C*. *parapsilosis*. Other studies have also found a correlation between *C*. *albicans* and *C*. *glabrata* with higher mortality, as well as lower mortality rates in cases of candidemia due to *C*. *parapsilosis* [11, 24, 31, 32, 35].

Another important finding of our study was the high frequency of fluconazole use, being the first choice in most cases. A recent guideline for the management of candidiasis recommended an echinocandin as initial therapy for candidemia [9], however this class of antifungal drugs is not yet very accessible due to its high cost [40]. Amphotericin B deoxycholate was the second most commonly used antifungal drug, however its lipid formulations are preferable because of its high toxicity [41], except in some specific cases [9].

Comparing our results using univariate and multivariate logistic regression analysis with the existing literature, we found other studies that have demonstrated an association between age, clinical condition (sepsis, septic shock, APACHE score) and mechanical ventilation with a higher mortality risk in patients with candidemia [30, 32, 35, 42, 43]. It is important to mention that hypotension is one of the criteria for the definition of septic shock [17] and it is also evaluated in the APACHE score, therefore its association with worse prognosis found in our study was expected; although we highlighted that its association as an independent risk factor had not yet been described.

Despite the low rate of antifungal resistance found in our study, there was a higher proportion of strains susceptible dose-dependent to fluconazole (20.5%), mainly among *C*. *glabrata* isolates (80%), consistent with the widely known lower susceptibility of *C*. *glabrata* to fluconazole [11]. These results indicate a greater probability of therapeutic failure if fluconazole is used, especially in cases of *C*. *glabrata* BSI.

Finally, our antifungal susceptibility profile corroborates with other studies conducted in Brazil and Latin America in general, where *Candida* spp. resistance to echinocandins and amphotericin B remains rare [39, 44].

In conclusion, we observed a high incidence of candidemia, displaying a tendency to increase over the 6 years of the study, as well as a high mortality rate, proving a nosocomial problem that deserves attention. We believe that our study contributed to the knowledge of the local epidemiology of candidemia and could be used to assist in the adoption of strategies to stratify patients at higher risk for developing candidemia and worse prognosis in low income regions of the globe, in addition to improve antifungal management (prophylaxis, empirical and definitive therapy) which has not been shown to be effective in the study hospital. We emphasize that this is the first study in Northeast Brazil that has made such a deep analysis in this regard, despite our limitations, mainly due to the nature of the study (retrospective and single center).

## Supporting information

S1 File Manuscript data set. (XLSX)
